# Prenatal diagnosis of caudal regression syndrome and omphalocele in a fetus of a diabetic mother

**DOI:** 10.11604/pamj.2017.27.128.12041

**Published:** 2017-06-16

**Authors:** Haifa Bouchahda, Houda El Mhabrech, Hechmi Ben Hamouda, Sobhi Ghanmi, Rim Bouchahda, Habib Soua

**Affiliations:** 1 Department of Gynecology Obstetric, University of Monastir, Tahar Sfar University Hospital, 5111 Mahdia, Tunisia; 2 Department of Radiology, University of Monastir, Maternal and Fetal Unit, Fattouma Bourguiba University Hospital,5000 Monastir, Tunisia; 3 Department of Neonatology, University of Monastir, Tahar Sfar University Hospital, 5111 Mahdia, Tunisia

**Keywords:** Caudal regression syndrome, prenatal diagnosis, maternal diabetes mellitus, prognosis

## Abstract

The caudal regression syndrome is defined as total or partial agenesis of the sacrum and lumbar spine, frequently associated with other developmental malformations (orthopedic, neurological, genito-urinary, gastrointestinal…). Prenatal diagnosis is possible through fetal ultrasound (US) and magnetic resonance imaging (MRI). A case of fetal caudal regression syndrome with omphalocele from a diabetic mother is presented, demonstrating the sonographic, MRI, CT and X-Ray features diagnostic. We will also discuss neonatal findings, risk factors and prognosis of this condition.

## Introduction

The caudal regression syndrome is a rare congenital malformation [1]. Defined as total or partial agenesis of the sacrum and lumbar spine, frequently associated with other developmental malformations (orthopedic, neurological, genito-urinary, gastrointestinal…). Prenatal diagnosis is possible through fetal ultrasound and MRI [1, 2]. The etiopathogenesis of this condition is not univocal [2], maternal diabetes appears to be among the major risk factors [3]. This anomaly is not lethal but the prognosis depends on the associated malformations. We report a case of this syndrome suspected at 28 weeks of gestation ultrasound and confirmed by fetal MRI.

## Patient and observation

**Figure 1:**
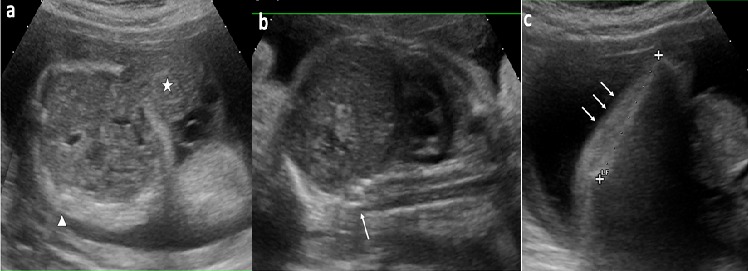
Obstetric ultrasound; a): axial section of the fetal abdomen, omphalocele with gray content (star), surrounded by a thin wall where passes the umbilical vein; The collar measures : 1.5 cm (dotted); b): in strict sagittal section of the fetus, abrupt interruption of the dorsal spine with no visualization of the lumbosacral and coccygeal segments (arrow); c): centered on the right lower limb, anomaly of the echogenicity of the lower limb with dedifferentiation of the musculoskeletal plans (arrows)

**Figure 2:**
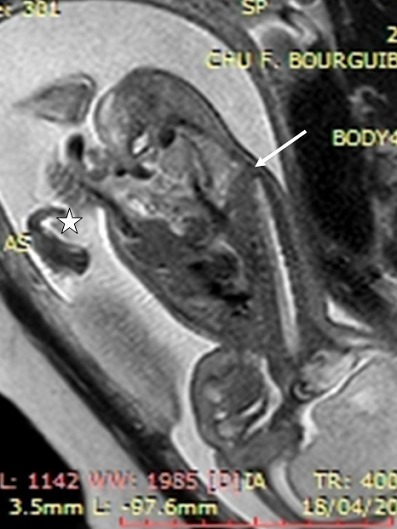
Fetal MRI in SE T2, in strict sagittal section confirming the agenesis of the lumbosacral spine (arrow); the omphalic sac is also visualized (star)

**Figure 3:**
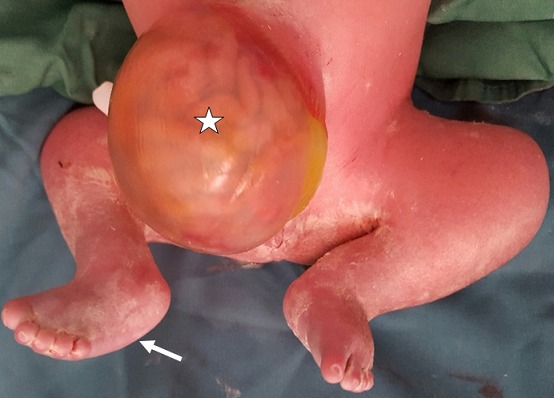
Photograph of the newborn: omphalocele containing digestive loops (star); appearance in frog of the two lower limbs with right clubbed foot (arrow)

**Figure 4:**
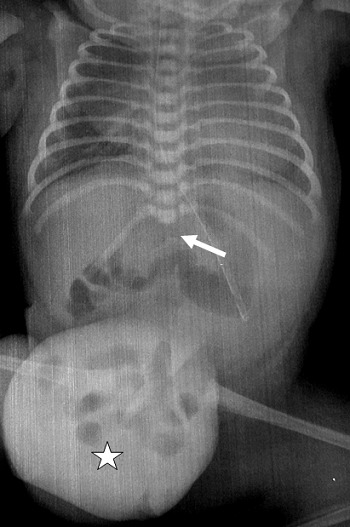
Postnatal thoracoabdominal X-ray: there are 12 dorsal vertebrae of normal morphology, with total agenesis of the lumbosacral spine (arrow); the omphalic sac containing digestive clarity

A 24-year-old woman, gravida 3, para 3, asthmatic for 13 years and diabetic with high dose of insulin for 2 years. The current unplanned pregnancy was poorly followed. The first ultrasound was performed at 28 weeks of gestation. It revealed an anterior medial mass of the anterior abdominal wall, well limited, surrounded by a thin membrane where passes the umbilical vein (Figure 1 a). It was an omphalocele containing small intestine with a collar measuring 1.5 cm. The insertion of the umbilical cord on the top of the omphalic sac was thick in relation to an over-developed Wharton´s jelly. It was associated with a short rachis limited only to the thoracic stage. Indeed, there was an agenesis of lumbar vertebrae, sacral and coccygeal on the axial (Figure 1 a) and sagittal sections (Figure 1 b). The lower limbs were frozen, of echo structure distinctly different from that of the upper limbs with bone segments little echogenic (Figure 1 c). The long bones (femurs and humerus) were shortened. The femur measured 3.92 cm and the humerus was 3.52 cm corresponding to an ultrasound term of 22 weeks of gestation. There was also a moderate ventriculomegaly, measured at 12 mm. A fetal MRI was then performed (Figure 2). Its objective was to study the spine and the spinal cord and to seek an etiology for ventriculomegaly. It confirmed the omphalocele and its content. It allowed a more precise spinal analysis by showing an interruption of the spine with agenesis of the lumbar, sacral and coccygeal segments. The medullary cord was visible until the end of the spine. The delivery was performed by Caesarean section at 40 weeks of gestation for breech presentation. The newborn, male, was hospitalized in neonatal resuscitation because of neonatal respiratory distress associated with a malformative syndrome. His Apgar was five in the first minute and then seven in the fifth minute. The clinical examination found a weight at 3600 g, a small size at 36 cm, and a macrocrania with a cranial perimeter at 38 cm. The omphalocele was found to be 10 cm in diameter (Figure 3). There was also a micromyelia of the two lower limbs, feet bots, paraplegia of the two lower limbs and peripheral axial hypotonia, a polypnea at 80 cycles/min and a generalized cyanosis. Cardiac auscultation was normal. The thoraco-abdominal radiograph showed cardiomegaly, digestive clarity in the omphalocele and lumbo-sacrococcygeal agenesis (Figure 4). The karyotype was normal (46, XY). The evolution was marked by the death after five hour of life by neonatal respiratory distress.

## Discussion

The lumbosacral agenesis also known as caudal regression syndrome is an abnormality of placement of the terminal part of the fetal spine occurring before the 9th week of development [1, 2] .Embryology it is either an early disappearance of the caudal appendix (exaggeration of a normal process) or an absence of development of the caudal appendix (caudal bud abortion). The consequence of the absence of the caudal bud is the approximation and fusion of the rudiments of the lower limbs up to the sirenomelia. The frequency of caudal regression syndrome is 0.1 to 0.4 per 10,000 births [2]. The sex ratio is estimated at 3 boys for a girl [4]. This condition is not described as a hereditary condition. Indeed, the risk of recidivism is very low [3]. The etiopathogenesis of caudal regression syndrome is multifactorial. Environmental factors and a genetic predisposition (mutation of the HLBX9 gene, CYP26A1, Wnt3a …) are incriminated [2, 3]. In our newborn, the karyotype was normal (46, XY) and the genetic study could not be carried out. The environmental factors are essentially maternal diabetes, early in utero exposure to toxins, certain infectious agents, excessive levels of retinoic acid, …[2]. Sixteen to twenty percent of fetuses with lumbosacral agenesis are from diabetic mothers as is the case with our patient. Maternal diabetes multiplies the risk of infection by 200[3]. However, this syndrome is not specific to diabetes and only 1% of diabetic mother babies may develop this type of malformation [4, 5].

Renshaw classified the spectrum of CRS into 5 types based on type of defect and articulation between bones. Type 1 has total or partial unilateral sacral agenesis. Type II has variable lumbar and total sacral agenesis and the ilia articulates with the sides of the lowest vertebra. Type III: has variable lumbar and total sacral agenesis and the caudal end plate of the lowest vertebra rests above fused ilia or an iliac amphiarthrosis. Type IV: has fusion of soft tissues in both lower limbs; and type V, also known as sirenomelia, has fused bones of lower limbs [6]. Our newborn seems to carry caudal regression syndrome type 4. Antenatal diagnosis can be suspected in the first trimester and confirmed in the second trimester of gestation. In the first trimester, lumbosacral agenesis will result in a decrease in cranio-caudal length [7]. During the second trimester, there was a lack of visualization of the sacrum and / or lumbar spine with abrupt interruption of the image of the rachis more or less extended during the study of the spine. The lower limbs, generally immobile, present a characteristic position: in “frog”, characteristic [7]. Their echostructure is also a peculiar testimony to the hypotrophy of the muscular-cutaneous masses. This was the case of our observation or we observed an anomaly of echogenicity of the musculoskeletal tissues of the lower limbs that can be explained by a neurological origin [8]. The morphological ultrasound is oriented towards the diagnosis and helps to count the other frequently associated malformations. There is a spectrum of malformations affecting the caudal end of the trunk ranging from isolated and partial agenesis of the sacro-coccygeal spine to more severe malformations. Urogenital, gastrointestinal malformations, respiratory diseases and congenital cardiac abnormalities may be associated. In our case, the omphalocele is the sign of major ultrasound call that led us to detect the other malformations as the amputation of the spine and the right clubfoot.

Fetal MRI makes it possible to confirm the diagnosis and to determine the level of the terminal medullary cone, which will be a major prognostic factor [9]. The spinal cord is usually dysplastic and the terminal cone is too high. Fetal MRI specifies the contents of the omphalic sac and eliminates the presence of other abnormalities of the neural tube notably encephalic ones. The prognosis of the caudal regression syndrome is depended on the prognosis of the associated malformations, the level of the medullary cone and the fetal repercussions of diabetes [1, 2, 10]. The neonatal death of our newborn was explained by neonatal respiratory distress in a diabetic mother´s baby. The prevention of lumbosacral agenesis is essentially based on good control of the carbohydrate balance in women with diabetes during the periconceptional period with multidisciplinary management of their pregnancy [4]. Compliance with the specifications of prenatal screening of the first and second trimester allows in utero radiological diagnosis and planning for neonatal care.

## Conclusion

The caudal regression syndrome is a closed spinal dysraphy associated with a wide variety of malformations on which the subsequent prognosis of newborns depends. The balance of glycemic figures in periconceptional women with diabetes, the genetic study of families with an index case and the respect of the specifications of the antenatal screening will probably offer the opportunity for a better management of this condition whose diagnosis is based on ultrasound and MRI.

## Competing interests

The authors declare no competing interest.
